# Prophylactic mesh placement for the PREvention of paraSTOmal hernias: The PRESTO systematic review and meta-analysis

**DOI:** 10.1371/journal.pone.0171548

**Published:** 2017-02-09

**Authors:** Frank Pianka, Pascal Probst, Anne-Valerie Keller, Daniel Saure, Kathrin Grummich, Markus W. Büchler, Markus K. Diener

**Affiliations:** 1 Department of General, Visceral and Transplantation Surgery, University of Heidelberg, Heidelberg, Germany; 2 Study Center of the German Surgical Society, University of Heidelberg, Heidelberg, Germany; 3 Institute of Medical Biometry and Informatics, University of Heidelberg, Heidelberg, Germany; University Hospital Oldenburg, GERMANY

## Abstract

**Background:**

Parastomal hernia (PH) is the most common complication after ostomy formation. Prophylactic mesh placement may be effective in reducing the rate of PH at the stoma site. The aims of this systematic review were to summarize the evidence with regard to the safety and effectiveness in comparison with the standard procedure without mesh placement and to identify important risk constellations.

**Method:**

A systematic literature search was performed in PubMed, EMBASE and the Cochrane library with no language or date restrictions. Randomized (RCTs) and non-randomized controlled trials (nRCTs) were included. The main outcomes of interest were PH (primary outcome) rate and stoma-related complications (secondary outcomes) such as stenosis or fistula. Statistical analysis included meta-analyses of pooled data and subgroup analyses.

**Results:**

Eleven trials (eight RCTs; three nRCTs) with a total of 755 patients were included. PH rate varied from 0% to 59% in the intervention and from 20% to 94% in the control group. RCTs showed a significant reduction of PH rate in the mesh group (OR 0.24; 95% CI 0.10 to 0.58, p = 0.034), whereas included nRCTs did not. No significant differences were observed in postoperative complication rates. Subgroup analyses showed superiority of non-absorbable meshes and sublay mesh positioning in open surgery.

**Conclusion:**

Prophylactic mesh placement is safe and reduces PH rate. A recommendation for prophylactic non-absorbable meshes in a sublay position can be made for patients undergoing open colorectal operations with end-ostomies. Further research endeavors should focus on patient-oriented outcomes, not only PH rate, with respect to tailored treatment in specific patient populations.

## Introduction

Parastomal hernia (PH), defined as an “incisional hernia related to an abdominal wall stoma”[1], is the most common complication of a permanent stoma. Its reported incidence varies widely, ranging from 4% to 56%, depending on the type of stoma, the length of follow-up and the use of imaging in diagnosis[2]. Although many PHs are asymptomatic[1], strangulation, obstruction and perforation are rare but serious complications[3–5]. PHs adversely affect patients’ quality of life as well as their ability to work[6]. Moreover, the rate of recurrence after PH repair is very high, ranging from 46.2% to 80.6% after suture repair[7–11], from 0% to 28.6% for mesh repair[12–18] and from 2.1 to 41.7% for laparoscopic repair[19–23]. The high prevalence of PH after stoma formation and the low success rate after PH repair demonstrate the importance of primary prevention of this surgical complication. Bayer et al. in 1986 were the first to describe mesh insertion at the time of primary stoma formation[24]. There are five systematic reviews evaluating this topic[2, 25–28]. Recently published randomized controlled trials (RCTs) render all of these reviews obsolete, however, and the important question of safety versus effectiveness has never been adequately addressed. Therefore, PHs remain an unresolved problem after stoma formation and are a substantial source of morbidity with considerable economic impact. Most PHs appear within the first few years after creation of the ostomy, but some develop as long as 20 years later[29]. To generate a larger sample size and to gain insight from trials with a longer follow-up period, systematic assessment of all available evidence is needed, including non-randomized comparative data. Only by this approach can the effectiveness of prophylactic mesh placement to prevent PHs be estimated more precisely. Thus, our preliminary literature search revealed additional RCTs and non-randomized controlled trials (nRCTs), which have not been included in previous analyses. Moreover, published systematic reviews have failed to implement important subgroup analyses such as the evaluation of mesh material used, operation technique, mesh positioning and stoma type.

The objective of this systematic review (working title: PREvention of paraSTOmal hernias–PRESTO) was firstly to summarize the available evidence on safety and effectiveness of prophylactic mesh placement to enable formulation of recommendations for future research endeavors and secondly to identify important risk constellations such as mesh type, mesh positioning and ostomy type. Our results can also serve as a reference for sample size calculation in future trials.

## Methods

After an external international peer review by clinical and methodological experts from the German Federal Ministry of Research and Education, the review protocol was approved and funding granted. The reporting of the extracted data complies with the “Preferred Reporting Items for Systematic Reviews and Meta-Analyses” (PRISMA) Statement[30] as outlined in a predefined protocol.

### Search strategy and sources of literature

A systematic literature search was conducted independently by two authors using validated methods of the Cochrane Collaboration[31]. The search was not restricted to specific languages or years of publication. The following databases were searched: The Cochrane Library database CENTRAL (Register of Controlled Trials), Medline and EMBASE. Search strategy was based on combinations of Medical Subject Heading (MeSH) terms and text words for each database, as shown in Box 1 for Medline.

**Box 1. Search strategy for the Medline database.**

(“parastomal hernia” OR “parastomal hernias” OR “parastomal herniation” OR “paracolostomy hernia” OR “paracolostomy hernias” OR “para-colostomy hernia” OR “para-colostomy hernias” OR stoma complication* OR “peristomal hernia” OR “peristomal hernias” OR “paraileostomy hernia” OR “paraileostomy hernias” OR “enterostomy site hernia” OR “enterostomy site hernias”) AND (mesh [tiab] OR “mesh placement” [tiab] OR “mesh implantation” [tiab] OR “prophylactic mesh” [tiab] OR prevention OR prophyl*)

The most recent systematic literature search was conducted in April 2016. Besides searches in the above-mentioned databases, reference lists of relevant articles were checked. The registry ClinicalTrials.gov was searched for registered and ongoing trials. Moreover, investigators and experts in the fields of colorectal and hernia surgery were consulted to help ensure that all trials relevant for this review had been identified.

Any discrepancies between the two main reviewers were discussed with a third reviewer (MKD) to reach consent and to decide inclusion for review.

### Study selection

All identified records were reviewed by two independent investigators. Trials were considered eligible for the systematic review if they met the following inclusion criteria: RCT or nRCT comparing mesh placement with conventional stoma formation in adult patients undergoing elective colorectal operation for malignant or inflammatory bowel diseases, with “parastomal hernia” reported as primary or secondary outcome. Animal trials and case reports were excluded, as were trials investigating stoma formation in an emergency setting. If the abstract suggested potential relevance, the full article was assessed for eligibility.

### Data extraction

Trial data were extracted based on predefined extraction forms independently by two reviewers. Any disagreement in the selection, extraction and or quality assessment process was resolved by consultation with a third member of the work group.

Baseline and surgical characteristics were extracted. Trial characteristics included title, authors, year of publication, journal, duration, design and sample size. Participants’ age, sex, ASA score[32], underlying disease and surgical procedures were the extracted baseline characteristics. Relevant complications and outcome variables included PH, stoma necrosis, reintervention due to mesh rejection, stoma fistula, stoma stricture, perioperative mortality and stoma site infections.

### Assessment of study quality

The methodological quality of included trials was assessed using the Cochrane Collaboration’s risk of bias tool to evaluate random sequence generation, allocation concealment, blinding of participants, personnel and outcome assessment, incomplete outcome data, selective reporting and other sources of bias. For each domain low, unclear or high risk of bias was assigned according to the Cochrane Handbook for Systematic Reviews of Interventions[31]. Visualization of the Cochrane risk of bias tool was performed with Review Manager (RevMan) version 5.3.5 (The Cochrane Collaboration, The Nordic Cochrane Centre, Copenhagen, Denmark).

### Statistical analysis

Statistical analysis was performed with R (Version 3.1.1, R Development Core Team 2015, Vienna, Austria). Meta-analyses were stratified into RCTs and nRCTs. The pooled results from the retrieved RCTs and nRCTs are presented as odds ratios (OR) with the associated 95% confidence intervals (95% CI). Differences with a P value < 0.05 were considered statistically significant. Following the recommendation of the Cochrane Collaboration we did not analyse the RCTs and nRCTs together.

Statistical heterogeneity among trials was evaluated by use of forest plots and the I^2^ statistic. In the case of substantial differences in methodological quality among individual trials or substantial clinical variability, sensitivity analyses were performed. Subgroup analyses were limited to the primary outcome and performed for data of RCTs and nRCTs separately. The following analyses were investigated: (1) absorbable vs. non-absorbable mesh, (2) intraperitoneal vs. sublay mesh position, (3) loop vs. end-ostomy, (4) open vs. laparoscopic approach, (5) malignant vs. both benign and malignant underlying disease. The subgroup analysis was done using the Cochran-Mantel-Haenszel test[33].

Concerning zero counts in our analysis, we used the Peto method[34], which according to the Cochrane Handbook “works well when intervention effects are small (odds ratios are close to one), events are not particularly common and the trials have similar numbers in experimental and control groups”. With this procedure it was possible to include trials with events in only one arm. Trials with no events are excluded from the analyses by giving them no weight, in accordance with the standard practice in meta-analysis of odds ratios. One reason for the exclusion of such trials is that they provide no indication of either the direction or the magnitude of the relative treatment effect. Nevertheless, when interpreting the results, one has to keep in mind that this procedure is conservative. We assumed that the observed estimates of treatment effect may vary across trials because of clinical differences in the treatment effect as well as sampling variability. Therefore, random-effects modelling was employed to account for clinical heterogeneity in the populations and surgical treatments. In the case of 10 or more included RCTs, testing for publication bias for the primary outcome was performed as recommended by the Cochrane Collaboration.

## Results

**Fig 1:**
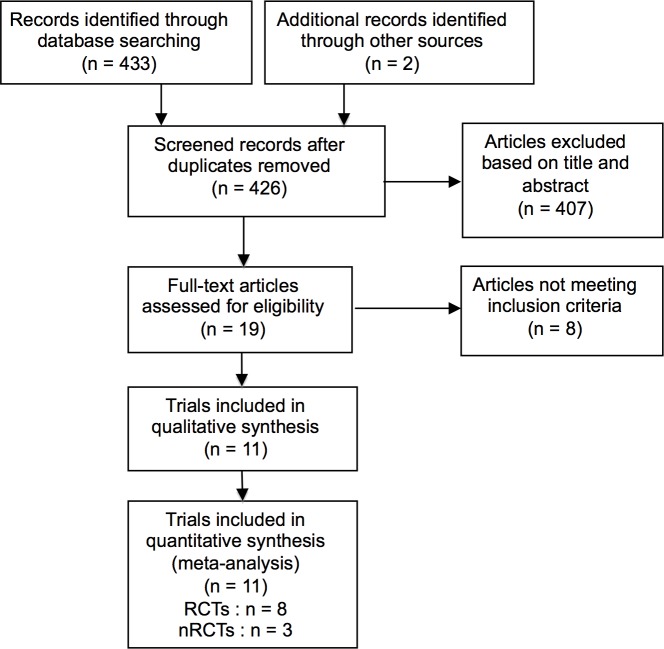
PRISMA flowchart.

The search yielded 435 returns in total. After removal of duplicates and screening of titles and abstracts, 19 articles remained. Full-text analysis led to exclusion of eight of these publications as they either did not investigate the intervention of interest or the study type was wrong (Fig 1). On clinicaltrials.gov, four trials were identified as ongoing, one of which was listed as “completed” but had not yet been published.

The 11 trials included in qualitative and quantitative analysis comprised eight RCTs[35–42] and three nRCTs[43–45] with a total of 755 patients. The articles were published between 2008 and 2015, ten in English and one in Chinese. All 11 reported PH as primary or secondary outcome.

One of the excluded trials investigated the intervention of interest and had been included in previously published systematic reviews[2, 25–28], but contained patients treated in an emergency setting, therefore meeting one of our exclusion criteria[46]. Trials including emergency operations were excluded due to the altered risk constellations, thus distorting patient population.

The characteristics of the included trials are presented in Table 1. All except two trials used a non-absorbable mesh in the intervention group. Hammond et al.[36] and Fleshman et al.[40] employed an absorbable/biodegradable acellular porcine collagen mesh.

**Table 1:** Characteristics of included trials.

Name	Study type	Study period	Sample size	Mesh type	Mesh position	Outcome assessment	Technique	Follow up	Cancer/benign
Hammond et al.[36] (2008)	RCT, single-centre	Not stated	20	Acellular porcine collagen matrix	Preperitoneal	Clinical, US	Open	6.5 (median)	both
Serra Aracil et al.[38] (2009)	RCT, single-centre	2004–2006	55	PG/PP	Sublay	Clinical, CT	Open	29 (median)	cancer
Lopez-Cano et al.[37] (2012)	RCT, single-centre	2007–2010	36	Polydioxanone-coated PP	IPOM	Clinical, CT	Laparoscopic	10.4 (median)	cancer
Cui et al.[35] (2009)	RCT, single-centre	2003–2005	60	PTFE	IPOM	Clinical, US, CT	Open	36 (mean)	cancer
Tarcoveanu et al.[39] (2014)	RCT, single-centre	2010–2011	42	PP	Sublay	Clinical, US	Open	20 (median)	cancer
Fleshman et al.[40] (2014)	RCT, multicentre	2010–2012	113	Acellular porcine collagen matrix	IPOM	Clinical, CT if suspicion of PH	Open, laparoscopic	24 (mean)	both
Lambrecht et al.[41] (2015)	RCT, two-centre	2007–2011	58	PP	Sublay	Clinical, CT	Open	40 (median)	cancer
Vierimaa et al.[42] (2015)	RCT, multicentre	2010–2013	70	PP/PVDF	IPOM	Clinical, CT	Laparoscopic	12 (mean)	cancer
Ventham et al.[44] (2012)	nRCT, retrospective, single-centre	2002–2010	41	PP	Sublay	CT	Open	13.3 (mean)	cancer
Jano et al.[43] (2014)	nRCT, prospective, single-centre	2003–2009	34	Two mesh types:	3D mesh, IPOM/sublay	Clinical, US, CT if suspicion of PH	Open, laparoscopic	12–72 (range)	cancer
				PP, PG/PP					
Nikberg et al.[45] (2015)	nRCT, prospective, single-centre	1996–2012	206	Two mesh types: PGL/PP,	Sublay	Clinical, CT	Open	31 (median)	cancer
				PET/PLA					

RCT randomized controlled trial, nRCT non-randomized controlled trial, PG poliglecaprone, PGL polyglactin, PP polypropylene, PTFE polytetrafluoroethylene, PVDF polyvinylidene fluoride, PET polyethylene terephthalate, PLA polylactic acid, 3D three-dimensional, IPOM intraperitoneal onlay mesh, US ultrasound, CT computed tomography, Follow up in month.

Permanent end colostomy was performed in all but two trials for malignant indications. Hammond et al. performed protective double loop ileostomy and Fleshman et al. end colostomy or ileostomy for various reasons, including inflammatory bowel diseases and other benign indications. Technical aspects of the operation were described in all 11 trials, six of which also gave information about the surgeon’s proficiency[37–39, 42, 44, 45]. Open surgery was performed in seven trials, two included both open and laparoscopic methods, and two featured only patients with laparoscopic surgical access. Meshes were placed in either sublay or intraperitoneal position; one trial used tube-like three-dimensional meshes consisting of an onlay and a sublay part[43].

Individual follow-up ranged from 1 to 202 months. Primary and secondary outcomes were assessed by clinical examination in all trials, with the exception that Ventham et al. described radiologic follow-up by computed tomography (CT) only, with retrospective assessment of secondary outcomes. Two groups described ultrasound[36, 39] in addition to clinical examination, five studies CT[37, 38, 41, 42, 45] and one trial both[35]. Two groups conducted ultrasound and/or CT[40, 43] when PH was suspected, otherwise performing a clinical examination only.

### Study population

The total study population comprised 409 patients in the control and 346 in the mesh groups. The baseline characteristics of all patients are displayed in Table 2. The characteristics of the control and mesh groups are not shown separately because reporting was limited. The sample sizes ranged from 20 to 206 patients. Differences in study populations were not significant in nine trials, Cui et al. and Tarcoveanu et al. did not provide population characteristics. All trials described malignant diseases as indication for primary surgery, and two trials also included patients with benign underlying diseases[36, 40].

**Table 2:** Population characteristics.

	RCT		nRCT		Total
	Control	Mesh	Control	Mesh	
	(n = 198)	(n = 212)	(n = 99)	(n = 143)	(n = 652)
**Age (years)**	60.5	63.5	66.6	69.0	64.9
**Sex f:m**	75:95	71:104	66:110	47:58	259:367
**BMI (kg/m**^**2**^**)**	26.1	25.9	27.0	27.0	26.5
**ASA**					
**I and II**	68	59	79	40	246
**III and IV**	41	48	56	31	176

Baseline characteristics of the study population (patients analyzed); incomplete patient numbers due to unavailability of data. BMI body mass index.

### Quality assessment and risk of bias

**Fig 2:**
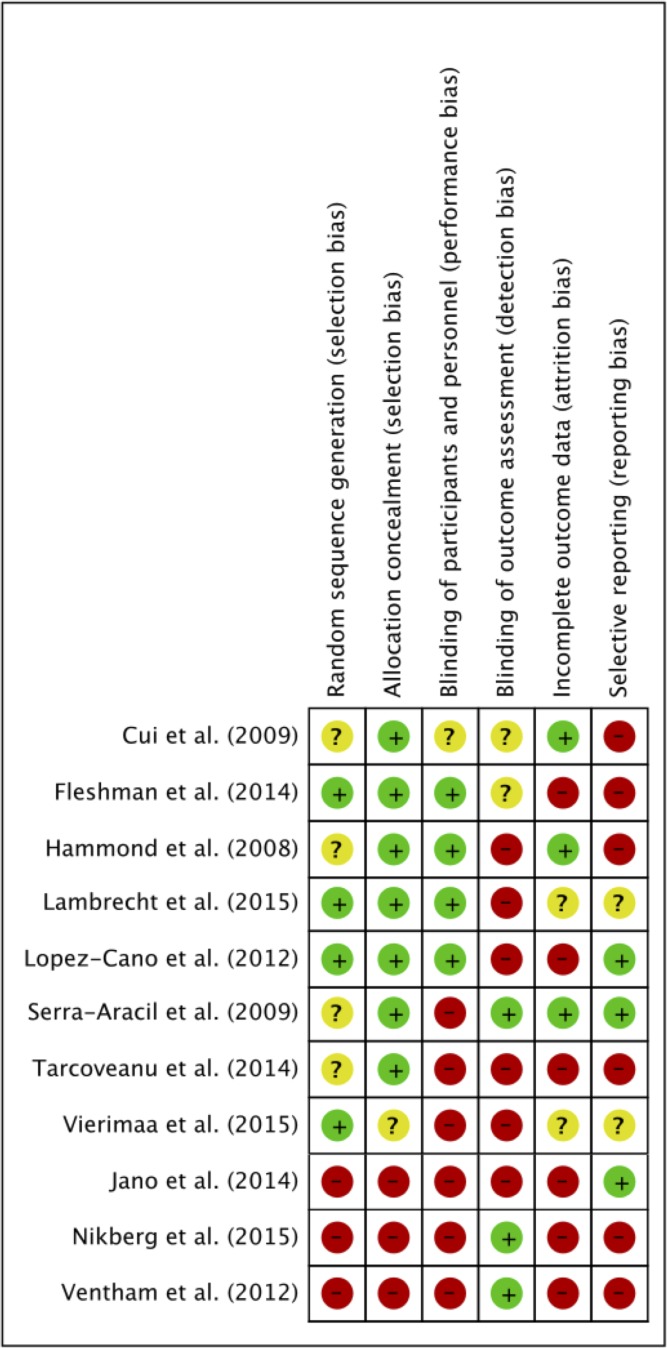
Risk of bias summary. Review authors' assessments of each risk of bias item for each included trial, presented as low (-), high (+) or unclear (?).

All 11 trials were analyzed for quality. The risk of bias is summarized in Fig 2.

Four RCTs described computer-based random sequence generation. The other four RCTs[35, 36, 38, 39] did not report on this domain, therefore risk of bias was classified as unclear. Allocation concealment was described by seven RCTs, five of which used sealed opaque envelopes and two a central randomization process. The three nRCTs were considered to have a high risk of selection bias.

Blinding of participants was adequately reported by four RCTs, blinding of outcome assessment by one RCT and two nRCTs. Two RCTs and two nRCTs did not describe any effort to implement blinding of participants or outcome assessment and were therefore assigned to high risk of bias.

Attrition bias was low in three RCTs as outcome reporting was complete. In six trials attrition bias was considered high, and the remaining two trials were classified as unclear in this respect.

Reporting bias was generally high. Four RCTs and two nRCTs were rated high due to reporting of previously unmentioned outcomes[35, 36, 39] or misleading reporting of analyzed patient groups. For example, Fleshman et al. reported a per-protocol and an intention-to-treat analysis strategy. Inexplicably, the intention-to-treat group was much smaller than the per-protocol group.

Ventham et al. discussed erroneous PH rates and bias was therefore classified as high.

### Quantitative analysis

The final meta-analysis included data of 410 randomized patients and 242 non-randomized patients.

#### Primary outcome: PH rate

**Fig 3:**
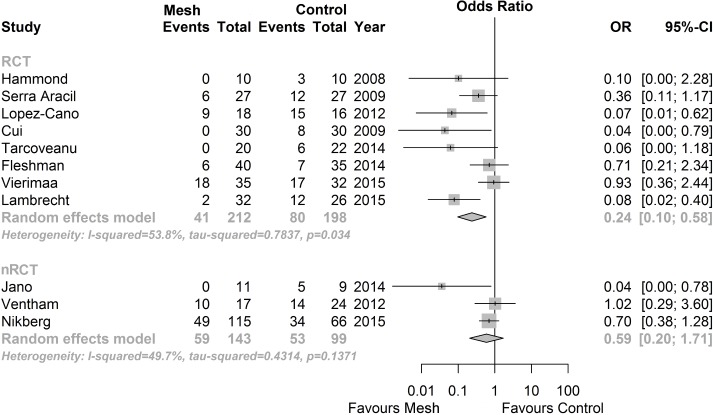
Forest plots for the primary outcome (parastomal hernia). Pooled data from eight RCTs and three nRCTs, with odds ratios (OR) and corresponding 95% confidence intervals (CI).

Overall PH rate was available in all eight RCTs[35–42] and all three nRCTs[43–45]. The RCTs showed a significantly reduced PH rate in the prophylactic mesh group compared to no mesh (OR 0.24; 95% CI 0.10 to 0.58; p = 0.034; I^2^ = 53.8%). Conversely, nRCTs did not show a significant superiority of prophylactic mesh placement (OR 0.59; 95% CI 0.20 to 1.71; p = 0.1371; I^2^ = 49.7%, see Fig 3).

#### Secondary outcomes

Reporting of secondary outcomes varied strongly across trials; it was generally weak in the nRCTs and more exhaustive in the RCTs. Analyses comparing investigated secondary outcomes in stomas constructed with and without prophylactic mesh placement are summarized in Table 3. In total, there were no significant differences between the two groups in either RCTs or nRCTs. No meta-analyses were performed for outcomes that were reported but had no events in either group.

**Table 3:** Meta-analyses of secondary outcomes.

Outcome	No. of trials	No. of patients	No. of events		Odds ratio [95% CI]	I^2^ [%]	P
			Mesh	No Mesh			
**RCTs**							
Stoma necrosis	6	348	7	10	0.65 [0.25; 1.71]	0	0.99
Reintervention due to mesh	4	213	2	0	1.81 [0.31; 10.68]	0	0.95
Stoma fistula	5	229	1	0	1.26 [0.23; 6.85]	0	0.99
Stoma stricture	5	302	6	2	1.75 [0.49; 6.35]	0	0.91
Stoma site infection	8	410	4	5	0.88 [0.28; 2.73]	0	0.99
Perioperative mortality	4	230	1	0	1.34 [0.21; 8.68]	0	0.96
**nRCTs**							
Stoma necrosis	3	242	0	1	0.52 [0.06; 4.44]	0	0.80
Reintervention due to mesh	3	242	0	0	0.87 [0.09; 8.62]	0	0.95
Stoma fistula	0	-	-	-	-	-	-
Stoma stricture	1	20	2	0	5.0 [0.21; 118.65]	0	0.99
Stoma site infection	2	61	2	1	2.13 [0.26; 17.65]	0	0.59
Perioperative mortality	1	181	2	1	1.15 [0.10; 12.93]	0	0.99

Meta-analyses of secondary outcomes among trials with odds ratios (OR) and corresponding 95% confidence intervals (CI) and heterogeneity (I^2^ index).

The stoma necrosis rate showed no significant difference between groups, with an OR of 0.65 (95% CI 0.25 to 1.71; p = 0.9882; I^2^ = 0%). Among the nRCTs only one event was reported in the control group (OR 0.52; 95% CI 0.06 to 4.44; p = 0.8036; I^2^ = 0%).

Stoma fistula was reported in five RCTs. The pooled OR was 1.26 (95% CI 0.23 to 6.85; p = 0.9893; I^2^ = 0%). No nRCT clearly reported this outcome.

Stoma stricture was stated in five RCTs and one nRCT. The OR was 1.75 (95% CI 0.49 to 6.35; p = 0.9114; I^2^ = 0%) for RCTs and 5.00 (95% CI 0.21 to 118.65; p > 0.9999; I^2^ = na) for nRCTs; no significant differences were found.

Stoma site infections were reported by all trials except Nikberg et al., with an OR of 0.88 (95% CI 0.28 to 2.73; p = 0.9901; I^2^ = 0%) for RCTs and 2.13 (95% CI 0.26 to 17.65; p = 0.5861; I^2^ = 0%) for nRCTs. Again, no significant differences were shown.

Four RCTs and one nRCT analyzed perioperative mortality, with one and two deaths in the experimental group respectively. The RCTs reported no perioperative deaths in the control group, the nRCTs one death. The OR was 1.34 (95% CI 0.21 to 8.68; p = 0.9594; I^2^ = 0%) for RCTs and 1.15 (95% CI 0.10 to 12.93; p > 0.9999; I^2^ = na) for nRCTs, with no statistically significant difference.

Reinterventions due to mesh-associated complications were reported in five of the eight RCTs and all three nRCTs. The pooled OR for RCTs was 1.81 (95% CI 0.31 to 10.68; p = 0.9481; I^2^ = 0%) and for nRCTs 0.87 (95% CI 0.09 to 8.62; p = 0.9521; I^2^ = 0%) with no significant difference.

### Subgroup analyses

The subgroup analyses were performed separately for RCTs and nRCTs. While the latter revealed no relevant results, the subgroup analyses for RCTs showed the following:

1. Absorbable vs. non-absorbable mesh. Two[36, 40] RCTs using absorbable mesh were compared with six[35, 37–39, 41, 42] RCTs using non-absorbable mesh. Insertion of non-absorbable mesh was associated with a significantly lower PH rate (OR 0.18; 95% CI 0.06 to 0.57; p = 0.023; I^2^ = 61.7%).
2. Intraperitoneal vs. sublay mesh position. Four[35–37, 42] RCTs using intraperitoneal mesh placement were compared with four RCTs[38–41] featuring sublay positioning of the mesh. The quantitative results significantly favored meshes placed in a sublay position (OR 0.25; 95% CI 0.09 to 0.75; p = 0.1165; I^2^ = 49.2%).
3. Loop vs. end ostomy. One[36] and seven[35, 37–42] RCTs, respectively, were available for the comparison of loop and end ostomy. We found significant superiority of the end ostomy group with regard to PH rate (OR 0.25; 95% CI 0.10 to 0.63; p = 0.0244; I^2^ = 58.7%).
4. Open vs. laparoscopic approach. Five trials[35, 36, 38, 39, 41] described an open, two trials[37, 42] a laparoscopic operation technique. One trial[40] reported both, open and laparoscopic operations. The results are significantly in favor of mesh placement in open operations (OR 0.16; 95% CI 0.07 to 0.37; p = 0.418; I^2^ = 0%).
5. Indication for surgery: malignant vs. both benign and malignant. Six[35, 37–39, 41, 42] trials with malignant indications for surgery were compared with two[36, 40] trials that contained patients with malignant and benign indications. The PH rate was significantly lower in the former group (OR 0.18; 95% CI 0.06 to 0.57; p = 0.023; I^2^ = 61.7%).

### Sensitivity analysis

For RCTs, sensitivity analyses were performed for different levels of risk of bias. A difference was found between trials with an unclear risk of bias (OR 0.21; 95% CI 0.08 to 0.54; p = 0.3932; I^2^ = 0%) and trials with a low risk of bias (OR 0.30; 95% CI 0.08 to 1.11; p = 0.0164; I^2^ = 70.8%) for the domain “random sequence generation”. Therefore, a potential selection bias is present. None of the other domains aroused suspicion of other forms of bias.

## Discussion

For a long time, prophylactic mesh placement as a measure to prevent PH was viewed with considerable scepticism. Surgeons feared infection and foreign body rejection. Although implantation of a mesh seemed the only efficient way to decrease the PH rate, translation of research findings into practice was hesitant; the first RCT on this issue was published as late as 2004.

Eight RCTs and three nRCTs with a total of 755 patients were qualitatively and quantitatively analyzed. The occurrence of PH was significantly decreased by prophylactic insertion of mesh. The PH rate in randomized patients undergoing prophylactic mesh placement was 19.3%, compared with 40.4% in the control group. With regard to safety, patients with prophylactic mesh placement had no more complications than patients without mesh. The placement of a mesh was especially beneficial in the subgroup with non-absorbable meshes and in patients with malignant diseases who were treated with an end ostomy in an open operation.

It is important to delineate our review from the previous systematic reviews on this topic. The two most recent comprehensive reviews were published in 2012[25, 26] and both analyzed the same three RCTs. A systematic review published by Fortelny et al. in 2015 focussed only on biological meshes and therefore included just two RCTs. Eight of the 11 trials included in our systematic review have appeared since 2012. Three newly published RCTs[40–42] added valuable data and power to our meta-analysis. Thus, we present here the largest meta-analysis with no restrictions concerning language or time of publication. The results of this systematic review are in line with the existing literature. However, for the first time a clear risk–benefit-based advantage has been shown in favor of prophylactic mesh placement for the prevention of PH.

The included trials feature several flaws that limit the strength of evidence. Generally, the quality of the analyzed trials was moderate and every trial had high risk of bias in at least one of the standard domains. Following the recommendations of the Cochrane Collaboration, publication bias was not estimated for RCTs/nRCTs as the number of each, included RCTs and nRCTs was below 10.

The discrepancy in findings between RCTs and nRCTs indicates a distortion of results by selection bias. In nRCTs, the choice for mesh placement lies with the surgeon and is often not bound to predefined algorithms. Therefore patients prone to the development of hernias have a higher probability of receiving a mesh. Also patients have a right to deny the placement of a mesh if they were informed about both procedures prior to the operation. If included in analysis, those patients potentiate bias. Furthermore the role of attrition bias becomes more important in nRCTs, especially in retrospective analyses, as patients that are lost to follow-up can be withheld from analysis, as study inclusion is generally not performed prospectively.

When regarding control groups throughout the studies, reporting of standardized techniques is limited but all RCTs described surgical methods and randomized allocation to one group. Postoperative patient care until discharge however is not thoroughly reported. Especially in retrospective studies, when postoperative care algorithms are hard to comprehend, this limitation might also increase the risk of bias.

The methodological limitations of the included studies involve the short follow-up (for RCTs 1–87 month), single centre design and limited blinding. Therefore late mesh-related complications may have been missed and limited external validity with high effect sizes in single centre RCTs are likely to increase bias. Interestingly the two included multicentre trials (Fleshman et al. and Vierimaa et al.) showed no significant difference in PSH rate. All included RCTs have a high or unclear risk of bias in at least one blinding item (patients and personnel or outcome assessment), resulting in an increased performance and detection bias.

Furthermore, the inhomogeneous reporting of stoma-related complications and the lack of data on quality of life limit the meaningfulness of the evidence. The fact that the clinical relevance of the diagnosed stomal hernias is unknown and may lead to recommending the right intervention to the wrong patient.

Recent literature also involves the comparison of stoma formation techniques. A systematic review, including 10 studies (2 RCTs and 8 nRCTs) on extraperitoneal (EPS) versus transperitoneal (TPS) colostomy formation[47], states the superiority of EPS with a risk ratio of 0.36 (95% CI, 0.21–0.62). The hernia rate in the EPS group was 6.3% (22 PSH of 347 patients) and 17.8% (125 PSH of 701 patients). On first sight the results seem to be revolutionary as PSH rates are 3 times lower in the EPS group than in our prophylactic mesh group (reminder: PSH rates in our RCT-meta-analysis are 19.3 vs 40.4% in the mesh and no mesh groups). But when looking closer, Kroese et al. conducted a combined meta-analysis of RCTs and retrospective studies, opposing the recommendation of the Cochrane collaboration buying a higher precision for undesirable uncertainty and unacceptable error. Furthermore is the sample size calculation of the two included RCTs not reported, one of which used a rather small sample size (18 patients in each group) with a total of 2 events (11% in the TPS group). The second RCT reported 128 patients and a total of 5 events (8% PSH in the TPS group). Both studies seem completely underpowered, minimizing its validity. Therefore the EPS-technique is indeed a promising alternative to mesh-augmentation but still needs adequate proof of its benefit.

### Consequences for clinical practice and future research

This systematic review of the most recently published trials shows that prophylactic mesh placement reduces the occurrence rate of PH and is a safe procedure. However, the limitations of the individual trials included forbid generalization of the findings to all patients. On the basis of the data presented here, placement of a prophylactic non-absorbable mesh in a sublay position for definitive end ostomy can be recommended in patients that undergo open colorectal operations. Future research endeavors should focus on selected patient groups, their individual risk–benefit balance and hard endpoints such as reduction of re-intervention rates and improvement of patient’s quality of life.

## Conclusion

Prophylactic mesh placement is safe and reduces the rate of PH. Placement of prophylactic non-absorbable meshes in a sublay position can be recommended for patients undergoing open colorectal operations with formation of a definitive end ostomy.

Clinical relevance of measured outcomes can vary distinctively to patient-oriented relevance, thus further research should investigate patient-oriented outcomes and not only PH rate with respect to tailored treatment in specific patient populations.

## Supporting information

S1 File PRISMA Checklist. The checklist for reporting systematic reviews, as recommended by the PRISMA Statement.
(DOC)

## Abbreviations

3D: Three-dimensional
95% CI: 95% confidence interval
BMI: Body mass index
CENTRAL: Cochrane Central Register of Controlled Trials
CI: Confidence interval
CT: Computed tomography
EMBASE: Excerpta Medica database
IPOM: Intraperitoneal onlay mesh
ITT: Intention-to-treat
MeSH: Medical Subject Heading
nRCT: Non-randomized controlled trial
OR: Odds ratio
PG: Poliglecaprone
PH: Parastomal hernia
PP: Polypropylene
PRISMA: Preferred Reporting Items for Systematic Reviews and Meta-Analyses
PTFE: Polytetrafluoroethylene
RCT: Randomized controlled trial
ROB: Risk of bias
